# Therapeutic potential of targeting the FLNA‐regulated Wee1 kinase in adrenocortical carcinomas

**DOI:** 10.1002/ijc.35239

**Published:** 2024-11-11

**Authors:** Emanuela Esposito, Giusy Marra, Rosa Catalano, Sara Maioli, Emma Nozza, Anna Maria Barbieri, Constanze Hantel, Guido Di Dalmazi, Sandra Sigala, Jens Geginat, Elisa Cassinotti, Ludovica Baldari, Serena Palmieri, Alessandra Mangone, Alfredo Berruti, Emanuele Ferrante, Giovanna Mantovani, Erika Peverelli

**Affiliations:** ^1^ Department of Clinical Sciences and Community Health University of Milan Milan Italy; ^2^ PhD Programme in Experimental Medicine University of Milan Milan Italy; ^3^ PhD Programme in Translational Medicine University of Milan Milan Italy; ^4^ Department of Endocrinology, Diabetology and Clinical Nutrition University Hospital Zurich (USZ) and University of Zurich (UZH) Zurich Switzerland; ^5^ Medizinische Klinik und Poliklinik III University Hospital Carl Gustav Carus Dresden Dresden Germany; ^6^ Division of Endocrinology and Diabetes Prevention and Care IRCCS Azienda Ospedaliero‐Universitaria di Bologna Bologna Italy; ^7^ Department of Medical and Surgical Sciences Alma Mater University of Bologna Bologna Italy; ^8^ Section of Pharmacology, Department of Molecular and Translational Medicine University of Brescia Brescia Italy; ^9^ Department of Surgery Fondazione IRCCS Ca' Granda Ospedale Maggiore Policlinico of Milan Milan Italy; ^10^ Endocrinology Unit Fondazione IRCCS Ca' Granda Ospedale Maggiore Policlinico of Milan Milan Italy; ^11^ Medical Oncology Unit ASST Spedali Civili di Brescia Brescia Italy; ^12^ Department of Medical & Surgical Specialties, Radiological Sciences & Public Health University of Brescia Brescia Italy

**Keywords:** adavosertib, adrenocortical carcinomas, cell cycle regulation, filamin A, therapeutic target, Wee1

## Abstract

Filamin A (FLNA) is poorly expressed in adrenocortical carcinomas (ACC) compared to adenomas (ACA). Its presence is associated to a less aggressive tumour behaviour, potentially due to its role in negatively regulating IGF1R signalling. Upregulation of G2/M Wee1 kinase was shown in FLNA‐deficient mouse neural progenitor cells, and it has been reported in several tumours. This study explored Wee1 expression in ACC and its regulation by FLNA, the effects of Wee1 inhibitor AZD1775, and the impact of FLNA on its efficacy in ACC cell lines and primary cells. Analysis of FLNA and Wee1 proteins revealed elevated Wee1 and reduced FLNA in ACC compared to normal adrenal gland. FLNA knockdown increased Wee1 protein in NCI‐H295R, MUC‐1, and in primary ACC cells. Higher p‐CDK1 and cyclin B1 were shown in FLNA‐silenced MUC‐1, while decreased Wee1, p‐CDK1 and cyclin B1 resulted after FLNA overexpression. Wee1 reduction was reverted by lactacystin treatment and FLNA transfection increased p‐Wee1 (Ser123), suggesting FLNA's role in targeting Wee1 for degradation. AZD1775 dose‐dependently reduced proliferation and viability in ACC cell lines and primary cultures, and it triggered MUC‐1 cell death. Similar effects were induced by Wee1 silencing. FLNA depletion augmented AZD1775's efficacy in reducing proliferation and potentiating apoptosis in MUC‐1 and primary cells. In conclusion, we demonstrated that FLNA regulates Wee1 expression by promoting its degradation, suggesting that low FLNA typical of ACC leads to increased Wee1 with consequent cancer cells growth. It proposes Wee1 inhibition as a new potential therapeutic approach for ACC, particularly for those lacking FLNA.

## Graphical abstract

What's new?

Adrenocortical carcinoma (ACC) is a rare but aggressive cancer of the adrenal gland. In many ACC cases, expression of the cytoskeletal protein filamin A (FLNA) is dramatically reduced; its presence in a tumour is associated with less aggressiveness. Here, the authors show that FLNA promotes degradation of the kinase Wee1, which is overexpressed in ACC. Treatment with a Wee1 inhibitor or genetic silencing of Wee1, they found, reduced viability and proliferation of ACC cells in culture. Inhibition of Wee1 could therefore be a potential treatment approach, particularly for tumours lacking FLNA.

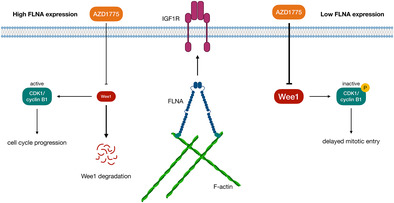

## 1 INTRODUCTION

Adrenocortical carcinoma (ACC) is a highly rare but extremely aggressive malignancy originating from the adrenal cortex, with an estimated annual incidence of 0.5–2 cases/million and a median overall survival of 3–4 years.^1^ Complete surgical resection represents the only curative treatment for localized disease, but the risk of recurrence is high (30–75%). However, more than 50% of ACC patients are diagnosed at an advanced or metastatic stage with a 5‐year survival <15%.^2^ Systemic chemotherapy with etoposide, doxorubicin, and cisplatin in combination with mitotane (EDP‐M scheme) constitutes the current standard‐of‐care for advanced/metastatic ACC,^3^ but its efficacy is limited and often results into toxic side‐effects.^4^ Therefore, the exploitation of novel treatment targets and therapies is urgently needed.

The molecular pathogenesis of ACC is partially understood so far.^5^, ^6^ Recently, we have provided evidence of a hugely reduced expression of the cytoskeletal protein filamin A (FLNA) in ACC compared to adenomas (ACA).^7^ Particularly, FLNA appeared to be a potential “protective” factor for ACC aggressiveness as its presence, although at low levels, was associated with a less aggressive tumour behaviour (lower ENSAT stage, Weiss score, and S‐GRAS score).^7^ One of the involved mechanisms is the ability of FLNA to repress the hyperactivated IGF2 signalling by downregulating the insulin‐like growth factor 1 receptor (IGF1R) in ACC cells.^8^ Indeed, despite being a major director of cytoskeletal architecture and dynamics, FLNA also functions as a scaffolding platform for about a hundred of different proteins, acting as a molecular hub that coordinates many signalling pathways.^9^, ^10^ In neural progenitor stem cells, FLNA has been reported to regulate the levels of Wee1 kinase protein,^11^ a key G2/M checkpoint gatekeeper mainly involved in controlling the timing of mitotic onset. Specifically, Wee1‐mediated inhibitory phosphorylation of cyclin‐dependent kinase 1 (CDK1) at Tyr15 residue keeps the CDK1‐cyclin B1 complex inactive, allowing DNA damage repair before mitotic entry.^12^ In the absence of DNA lesions, mitotic events are initiated by Cell Division Cycle 25C (Cdc25C), which turns the complex on by dephosphorylating CDK1.^12^ At this stage, phosphorylation of Wee1 at Ser123 by activated CDK1 protein gives rise to a cascade of events that finally terminates with Wee1 degradation by the ubiquitin proteasome system.^13^ An additional role of Wee1 at S‐ phase checkpoint has recently emerged, where it protects replication forks from DNA damages. Increased CDK1 activity due to Wee1 inhibition delays replication fork progression, leading to replication stress and loss of genome integrity.^14^, ^15^

Upregulation of Wee1 is common to several cancers and it usually correlates to a poor prognosis.^16^, ^17^, ^18^, ^19^ Moreover, Wee1 overexpression has been frequently reported in *TP53*‐mutated tumours with deregulated G1/S, which strongly rely on the G2/M checkpoint for DNA repair and survival.^20^ Therefore, Wee1 has become an attractive target for cancer therapy, as its inhibition forces tumour cells with unrepaired DNA damage to death through the processes of replicative or mitotic catastrophe.^21^, ^22^ To date, AZD1775 is the only Wee1 inhibitor in clinical development. Despite p53‐deficient cancer cells seem to be preferentially sensitized by Wee1 inhibition,^19^, ^20^, ^21^ the correlation between AZD1775 sensitivity and *TP53* mutational status is still highly controversial.^23^, ^24^, ^25^ Many phase I/II studies reported the efficacy of AZD1775 in different tumour types, either as monotherapy or in combination with different DNA‐damaging agents.^26^, ^27^, ^28^ Recently, AZD1775 anti‐tumour effects on ACC cells have been tested in a large‐scale drug screening.^29^ However, up to now, neither Wee1 expression nor the effects of its inhibitor AZD1775 has been explored in ACC thoroughly.

Aims of the present study were to elucidate the role of Wee1 in promoting tumour progression in ACC, to test the anti‐tumour effects of Wee1 inhibition, and to investigate the role played by FLNA in regulating Wee1 expression and the response to its specific inhibitor AZD1775 in human ACC cell lines and in patient‐derived primary cultured ACC cells.

## 2 MATERIALS AND METHODS

### 2.1 ACC cell cultures and adrenal tissues

Three human ACC cell lines were used. NCI‐H295R (CVCL_0458), derived from a primitive ACC,^30^ was obtained from the American Type Culture Collection (ATCC, Rockville, MD). NCI‐H295R cells were cultured in DMEM/F‐12, HEPES media (Gibco, Life Technologies, Carlsband, CA), supplemented with 1% ITS + Premix (Corning, NY), 2.5% Nu‐Serum (Corning, NY), and 1% penicillin–streptomycin (Lonza Group AG, Basel, CH). MUC‐1 (CVCL_C4KG), established from an ACC neck metastasis,^31^ were cultured with Advanced DMEM/F12 media (Gibco, Life Technologies, Carlsband, CA), supplemented with 10% FBS and 1% penicillin–streptomycin. TVBF‐7 (CVCL_C4KR), derived from a perirenal lymph‐node ACC metastasis,^32^ were maintained in Advanced DMEM/F12 media, supplemented with 10% FBS, 1% amphotericin B and 2 mM glutamine (Gibco, Life Technologies, Carlsband, CA), and 1% penicillin–streptomycin. All human ACC cell lines have been authenticated using STR profiling. All experiments were performed with mycoplasma‐free cells. Fresh ACC tumour tissues from two patients were dissociated to obtain primary cell cultures. Tumours were mechanically and enzymatically dissociated with 2 mg/ml collagenase type IA (Merck, Darmstadt, Germany) dissolved in DMEM (Sigma‐Aldrich, St. Louis, MO) at 37°C for 2 h. Undigested material was removed by a 100 μm cell strainer (BD Transduction Laboratories, Lexington, UK). Dispersed ACC cells were cultured in DMEM supplemented with 20% FBS and 1% penicillin–streptomycin. Cells were kept at 37°C in a humified atmosphere with 5% CO_2_. Frozen tissue samples were also used (ACC, *n* = 6; ACA, *n* = 8; NAG [tumour‐free normal adrenal glands derived from surgery of patients with kidney cancer, or peritumoral tissue derived from adrenalectomy], *n* = 8), from which protein samples were extracted and analysed by Western blotting. Tissue samples were mechanically disaggregated by using scalpels, lysed in 100 μl of ice‐cold cell lysis buffer (Cell Signaling Technology, Danvers, MA) with the addition of protease inhibitors (Roche Holding AG, Basel, SW), incubated on ice for 10 min, and then centrifuged at 13.000 rpm for 10 min at 4°C. The supernatant containing protein extracts was then transferred into a new tube and stored at −20°C. Proteins were quantified by Pierce BCA Protein Assay Kit (Thermo Fisher Scientific, Waltham, MA).

### 2.2 Chemical reagents

AZD1775 was purchased from TargetMol Chemicals Inc. (Boston, MA). The powder was dissolved in sterile dimethyl sulfoxide (DMSO) to prepare a stock solution of 1 mM. Lactacystin (Sigma‐Aldrich, St. Louis, MO) was used at a concentration of 10 μM.

### 2.3 FLNA and Wee1 silencing

Cells were seeded in 6‐well plates at a density of 3.0 × 10^5^ (NCI‐H295R, TVBF‐7, primary cells) and 1.5 × 10^5^ (MUC‐1) cells/well, or in 96‐well plates at a density of 1.4 × 10^4^ (NCI‐H295R, primary cells), 0.6 × 10^4^ (MUC‐1), and 2 × 10^4^ (TVBF‐7) cells/well in complete medium. The day after, cells were transfected using a human SMARTpool of FLNA or Wee1 predesigned small interfering RNAs (siRNA) purchased from Dharmacon (GE Healthcare Life Sciences, Chicago, IL), using Lipofectamine RNAiMAX (Thermo Fisher Scientific, Waltham, MA) as a transfection reagent. In each experiment, a negative control siRNA (non‐targeting sequence without a significant homology to the sequence of human, mouse, or rat transcripts) was used. For each experiment, silencing efficiency was checked by Western blot analysis, and only experiments with at least 70% silencing efficiency were considered.

### 2.4 FLNA transfection

MUC‐1 cells were seeded in 6‐well plates at a density of 1.5 × 10^5^ cells/well in complete medium. The day after, cells were transfected with pcDNA3‐Myc expression vector coding for myc‐tagged FLNA (Addgene, Watertown, MA) for 72 h using Lipofectamine 2000 (Invitrogen, Thermo Fisher Scientific, Waltham, MA) as transfection reagent. For each experiment, mock‐transfected cells (treated with the corresponding amount of Lipofectamine 2000 only) were used as negative control, and transfection efficiency was monitored by Western blot analysis by using a Myc‐tag antibody (Cell Signaling Technology, Danvers, MA).

### 2.5 Western blot

Cells were seeded in 6‐well plates at a density of 3.0 × 10^5^ (NCI‐H295R, TVBF‐7, primary cells) and 1.5 × 10^5^ (MUC‐1) cells/well, or in 96‐well plates at a density of 1.4 × 10^4^ (NCI‐H295R, primary cells), 0.6 × 10^4^ (MUC‐1) and 2 × 10^4^ (TVBF‐7) cells/well in complete medium for Western blot analysis. After extraction, total proteins were quantified by Pierce BCA Protein Assay Kit, separated on SDS/polyacrylamide gels, and transferred to a nitrocellulose filter. Wee1 antibody (Santa Cruz Biotechnology, Dallas, TX) and phospho‐Wee1(Ser123) (Bioss Antibodies, Woburn, MA) were diluted 1:100 and 1:200. Total‐FLNA (Abnova, Tapei, TW), total‐CDK1, phospho‐CDK1 (Tyr15) and cyclin B1 (Immunological Sciences, Rome, IT), and Myc‐tag, were used at 1:1000. Primary antibodies were incubated overnight at 4°C, while anti‐mouse/rabbit secondary antibodies (Cell Signaling Technology, Danvers, MA) were incubated at room temperature for 1 h at 1:2000. GAPDH antibody (Ambion, Thermo Fisher Scientific, Waltham, MA) was used at 1:4000 for 1 h at room temperature. Chemiluminescence was detected using ChemiDOC‐IT Imaging System (UVP, Upland, CA), and densitometrical analysis was performed with NIH ImageJ software. The detection of phosphorylated proteins was normalized on total proteins, and GAPDH was used as housekeeping.

### 2.6 Quantitative Real‐Time PCR

Total RNA from MUC‐1 cells was isolated through the RNeasy Mini Kit (Quiagen, Hilden, Germany), and 1 μg of extracted RNA was reverse‐transcribed with RevertAid H Minus First Strand cDNA Synthesis Kit (Thermo Fisher, Scientific, Waltham, MA). qRT‐PCR was carried out using the SsoFast EvaGreen Supermix (Bio‐Rad Laboratories, Hercules, CA) in a QuantStudio 3 Real‐Time PCR System (Applied Biosystems, Thermo Fisher Scientific, Waltham, MA). For human Wee1 and FLNA, specific primers were designed. GAPDH was used as housekeeping. Data were analysed with QuantStudio Design & Analysis Software v1.5.1, using the ΔCt method.

### 2.7 Cell proliferation assay

Cell proliferation was determined by colorimetric measurement of 5‐bromo‐2‐deoxyuridine (BrdU) incorporation during DNA synthesis in proliferating cells (Roche Holding AG, Basel, SW). Cells were seeded in 96‐well plates at a density of 1.4 × 10^4^ (NCI‐H295R, primary cells), 0.6 × 10^4^ (MUC‐1), and 2 × 10^4^ (TVBF‐7) cells/well in starved medium. The day after, cells were treated with AZD1775 for 72 h in complete medium. For FLNA and Wee1 gene silencing, cells were first transfected with the specific siRNA before being treated with AZD1775. Following stimulation, BrdU incorporation was allowed for 2 h (NCI‐H295R, MUC‐1, TVBF‐7) or 24 h (primary cells), and the assay was performed according to the manufacturer's instruction.

### 2.8 Cell viability assay

Cell viability was assessed by 3‐(4,5‐dimethyl‐2‐thiazol)‐2,5‐diphenyl‐2H‐tetrazolium bromide (MTT) dye reduction assay according to the manufacturer's protocol (Sigma‐Aldrich, St. Louis, MO). NCI‐H295R, MUC‐1 and TVBF‐7 were seeded in 96‐well plates at a density of 1.4 × 10^4^, 0.6 × 10^4^, and 2 × 10^4^ cells/well, respectively, in starved medium. After 72 h treatment with AZD1775, MTT (5 mg/ml in DMSO) was added to the cells in DMEM without phenol red supplemented with 10% FBS and incubated at 37°C for 4 h to allow the formation of purple‐coloured formazan crystals. The supernatant was then discarded and 100 μl of DMSO was added to dissolve formazan. Absorbance was read at 560 nm using the VICTOR Nivo Plate Reader (PerkinElmer Inc., Waltham, MA).

### 2.9 Cell apoptosis assay

MUC‐1 cells were seeded in 6‐well plates at the density of 1.5 × 10^5^ cells/well in complete medium. The day after, they were treated with AZD1775 for 72 h. For FLNA and Wee1 gene silencing, cells were first transfected with the specific siRNA before being treated with AZD1775. The Pacific Blue Annexin V/SYTOX AADVanced Apoptosis Kit (Invitrogen, Thermo Fisher Scientific, Waltham, MA) was used to analyse apoptosis by flow cytometry. Briefly, cells were harvested and washed with ice‐cold PBS, then stained and processed according to manufacturer's instruction. Unlabelled cells were used as negative control. Cells were acquired by FACSCanto II (BD Biosciences), and data were collected and analysed using FlowJo v10.9 software (FlowJo, LLC). Viable cells (Annexin V−/SYTOX−), early apoptotic (Annexin V+/SYTOX‐), late apoptotic (Annexin V+/SYTOX+), and necrotic (Annexin V‐/SYTOX+) are represented by the lower left, lower right, upper right, and upper left quadrants of flow cytometric plots, respectively.

### 2.10 Cell cycle analysis

MUC‐1 cells were seeded in 6‐well plates at the density of 1.5 × 10^5^ cells/well in complete medium. The day after, they were treated with AZD1775 for 72 h. After incubation, cells were harvested and washed with cold PBS, then fixed with pre‐cooled 70% ethanol for 30 min at 4°C. After washing twice in PBS, cells were resuspended with 1 ml PBS and incubated with propidium iodide (40 μg/ml) and RNase (100 μg/ml) for 30 min at room temperature protected from light. Cell cycle distribution was determined by using a FACSCanto II, and data were collected and analysed using FlowJo v10.9 software.

### 2.11 Immunofluorescence microscopy

3.0 × 10^4^ MUC‐1 cells/well were plated on 13‐mm‐poly‐L‐lysine coated coverslips in 24‐well plates. After 6 h stimulation with AZD1775, cells were fixed with 4% paraformaldehyde (Sigma‐Aldrich, St. Louis, MO) for 10 min at room temperature. After being washed with PBS, blocking buffer was used (5% FBS, 0.3% Triton X‐100, in PBS) for 1 h, and then cells were incubated overnight at 4°C with anti‐histone H2AX (1:400, Active Motif Inc., CA) antibody. The anti‐rabbit Alexa Fluor‐488‐conjugated secondary antibody (1:500, Thermo Fisher Scientific, Carlsbad, CA) was added at room temperature for 1 h. Antibody Diluent Reagent Solution (Life Technologies, Thermo Fisher Scientific, Carlsbad, CA) was used to dilute all antibodies. Coverslips were mounted on glass slides with EverBrite Hardset Mounting Medium with DAPI (Biotium, Fremont, CA) and analysed by fluorescence microscopy (Axio Vert.A1, Zeiss). Negative control coverslips were incubated with primary antibody only. Quantification of γ‐H2AX foci was performed by manual counting of individual foci within single nuclei, and at least 100 nuclei cells were scored for each experiment.

### 2.12 Statistical analysis

All statistical analyses were performed using GraphPad Prism 10.1.1 (GraphPad Software Inc., San Diego, CA). Data were summarized as the median with interquartile range (IQR) and were compared using non‐parametric tests. The differences between two groups were assessed using the Wilcoxon matched‐pairs signed rank test or Mann–Whitney test for paired and unpaired group of samples, respectively. The differences between multiple groups were assessed using the Friedman test or Kruskal–Wallis test with Dunn's post‐hoc test for paired and unpaired group of samples, respectively. A *p*‐value <0.05 was considered as statistically significant.

## 3 RESULTS

### 3.1 ACC, but not ACA, express lower FLNA but higher Wee1 protein compared to normal adrenal gland

**FIGURE 1:**
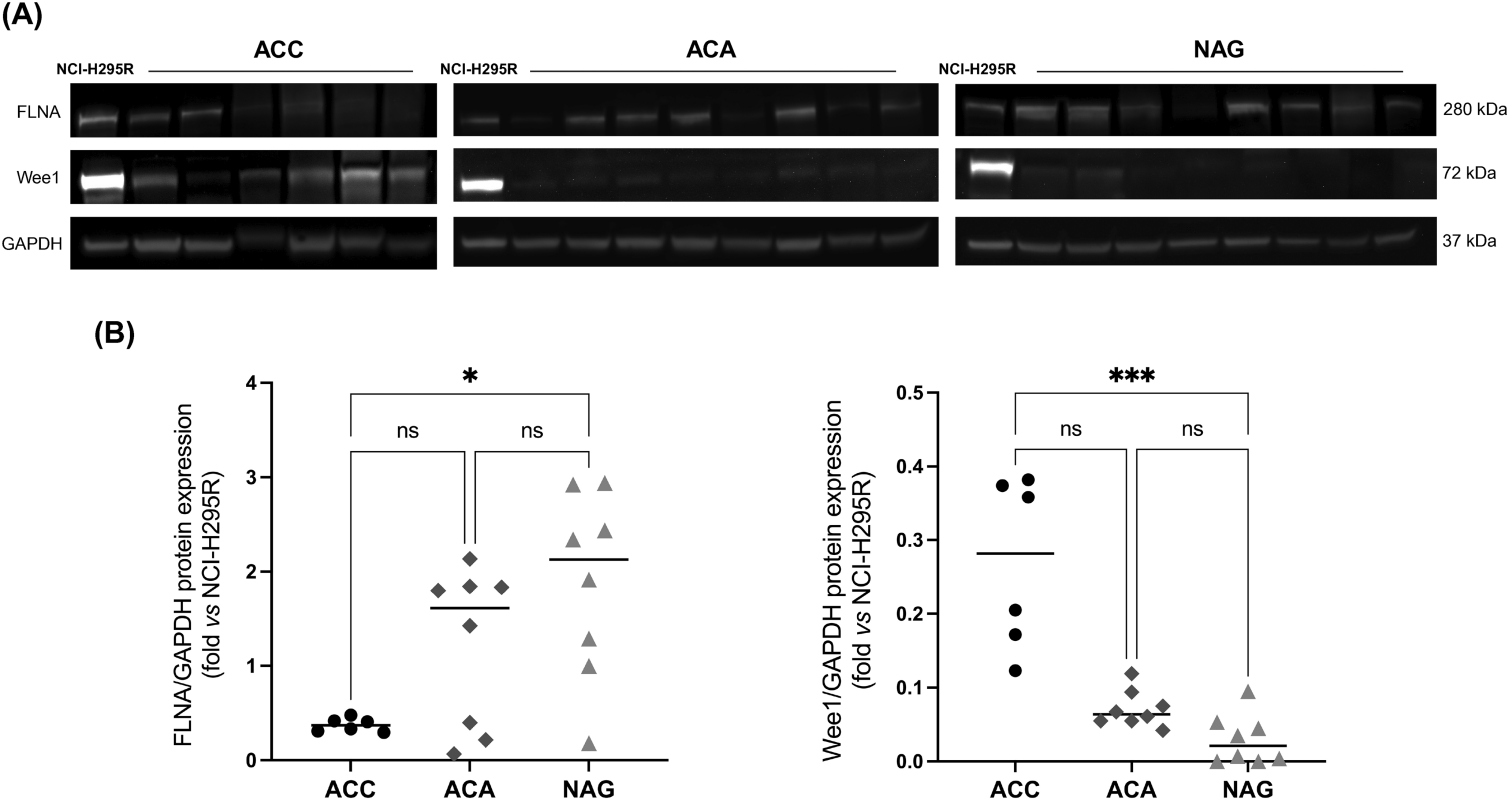
Differential FLNA and Wee1 protein expression in ACC, ACA, and NAG. (A) Representative immunoblots of FLNA, Wee1 and GAPDH expression in 6 patient‐derived ACC, 8 ACA, and 8 NAG. (B) The graphs show densitometric analysis of FLNA and Wee1 expression normalized to GAPDH and expressed as fold over NCI‐H295R. NCI‐H295R cell lysates were included to normalize all blots to the same control sample. Horizontal bars represent median. *p < 0.05; ***p < 0.001. Kruskal–Wallis multiple comparison test with Dunn's post‐hoc test.

We evaluated FLNA and Wee1 expression in 6 patients‐derived ACC, 8 ACA, and 8 normal adrenal gland (NAG) tissue samples by Western blot analysis. Interestingly, we found that ACC express, as expected, significantly lower FLNA protein levels compared to NAG (0.37[0.13] vs 2.13[1.73], *p* < 0.05) but markedly higher Wee1 (0.28[0.21] in ACC vs 0.02[0.05] in NAG, *p* < 0.001; Figure 1A,B). Conversely, no statistically significant difference in the expression of both FLNA and Wee1 proteins was reported in ACA, either when compared to NAG or ACC (Figure 1B). However, no correlation between FLNA and Wee1 expression levels was reported in ACC, ACA, and NAG.

### 3.2 FLNA silencing and overexpression induced opposite effects on Wee1 protein expression in MUC‐1 cells and in one patient‐derived ACC primary cultured cells

**FIGURE 2:**
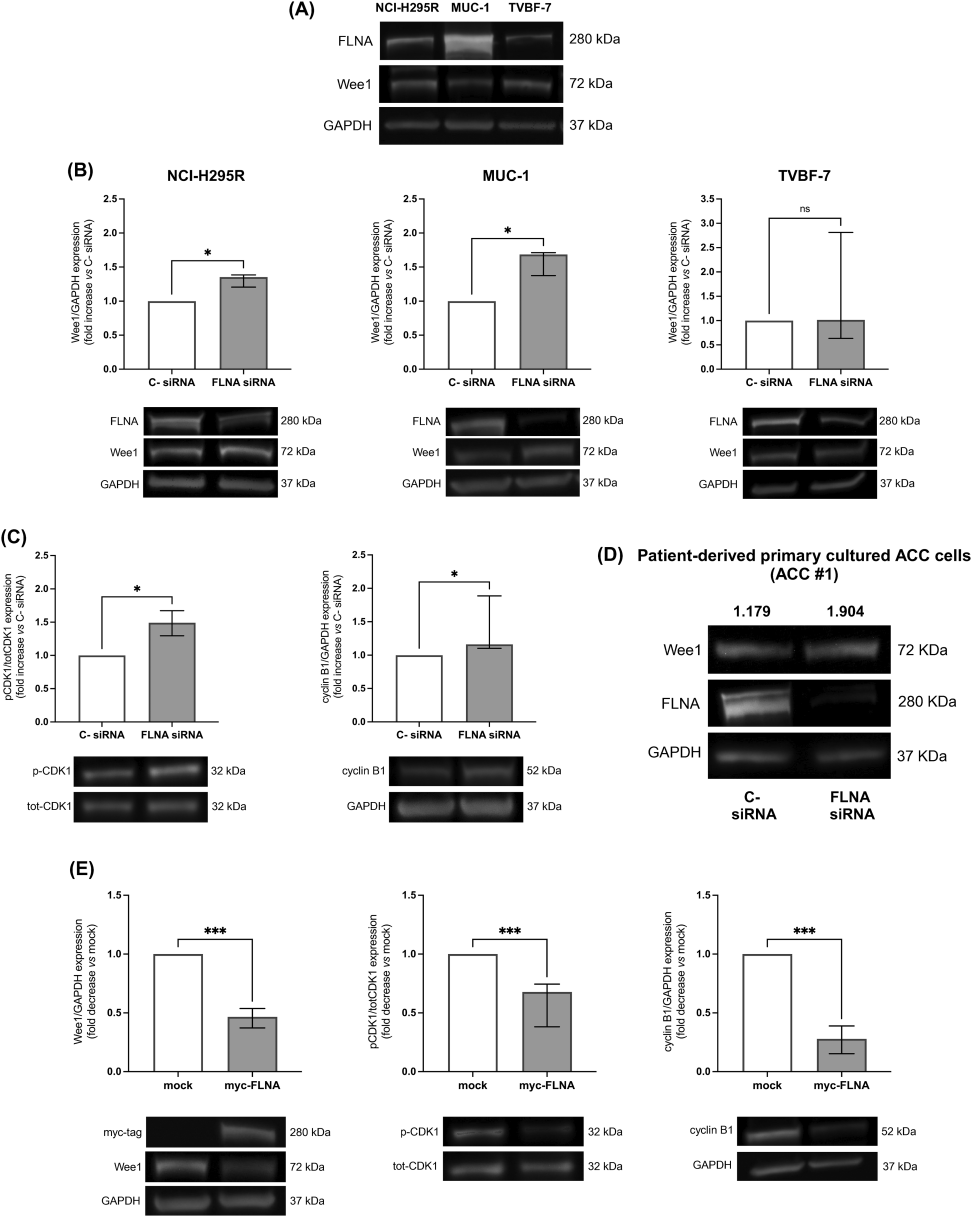
Expression of FLNA and Wee1 in human ACC cell lines, and effects of FLNA silencing and overexpression on Wee1 protein levels. (A) Protein expression levels of FLNA and Wee1 in NCI‐H295R, MUC‐1, and TVBF‐7 cell lines. Representative immunoblots of FLNA and Wee1 expression normalized to GAPDH are shown. (B) NCI‐H295R, MUC‐1, and TVBF‐7 cell lines were transfected with FLNA siRNA or negative control (C‐) siRNA for 6 days. The graphs show densitometric analysis of Wee1 normalized to GAPDH (median and IQR of at least 3 independent experiments, respectively). Representative immunoblots are shown. *p < 0.05 vs C‐ siRNA. Mann–Whitney test. (C) MUC‐1 cells were transfected with FLNA siRNA or negative control (C‐) siRNA for 6 days. The graphs show densitometric analysis of phosphorylated CDK1 (Tyr15) normalized to total CDK1 (median and IQR of at least 3 independent experiments), and of cyclin B1 normalized to GAPDH (median and IQR of at least 3 independent experiments). Representative immunoblots are shown. *p < 0.05 vs C‐ siRNA. Mann–Whitney test. (D) Patient‐derived primary cultured ACC cells (ACC #1) were transfected with FLNA siRNA or negative control (C‐) siRNA for 6 days. The values above immunoblot images indicate densitometric analysis of Wee1 normalized to GAPDH. (E) MUC‐1 cells were transiently transfected with myc‐tagged FLNA plasmid for 72 h. Mock‐transfected cells were used as negative control. The graphs show densitometric analysis of Wee1 and cyclin B1 normalized to GAPDH (median and IQR of at least 3 independent experiments, respectively), and phosphorylated CDK1 (Tyr15) normalized to total CDK1 (median and IQR of at least 3 independent experiments). Representative immunoblots are shown. ***p < 0.001 vs mock. Mann–Whitney test.

FLNA and Wee1 expression was then assessed in three different human ACC cell lines, namely NCI‐H295R, MUC‐1, and TVBF‐7. Both proteins were expressed in all of them, at variable levels (Figure 2A). To test a possible causal relationship between low FLNA levels detected in ACC and high Wee1 expression, we silenced FLNA in all cell lines. Our data showed that FLNA silencing significantly augmented Wee1 protein levels in NCI‐H295R (1.35‐fold[0.18], *p* < 0.05) and MUC‐1 (1.68‐fold[0.33], *p* < 0.05), but not in TVBF‐7 cells (Figure 2B). The efficiency of silencing was higher in MUC‐1 cells (−86.5 ± 9.3% vs C‐ siRNA) compared to that in NCI‐H295R (−51 ± 11.5% vs C‐ siRNA) and TVBF‐7 (−62 ± 25.2% vs C‐ siRNA). Therefore, MUC‐1 cell line was selected to further investigate FLNA role in Wee1 regulation. Interestingly, in MUC‐1 silenced for FLNA we also reported a significant increase in the expression of phosphorylated CDK1 and cyclin B1 (1.49‐fold[0.37] and 1.16‐fold[0.78], *p* < 0.05, respectively), suggesting a boost in Wee1 activity (Figure 2C). According to data obtained on cell lines, a higher Wee1 protein expression was also shown after FLNA silencing in one patient‐derived primary cultured ACC cells (1.61‐fold Wee1 increase vs C‐ siRNA; Figure 2D). Conversely, FLNA overexpression in MUC‐1 significantly decreased Wee1, phosphorylated CDK1 and cyclin B1 (0.46‐fold[0.16], 0.68‐fold[0.36], and 0.28‐fold[0.24], *p* < 0.001 vs mock‐transfected cells, respectively) (Figure 2E).

### 3.3 In MUC‐1 cells, FLNA directs Wee1 to proteasomal degradation

**FIGURE 3:**
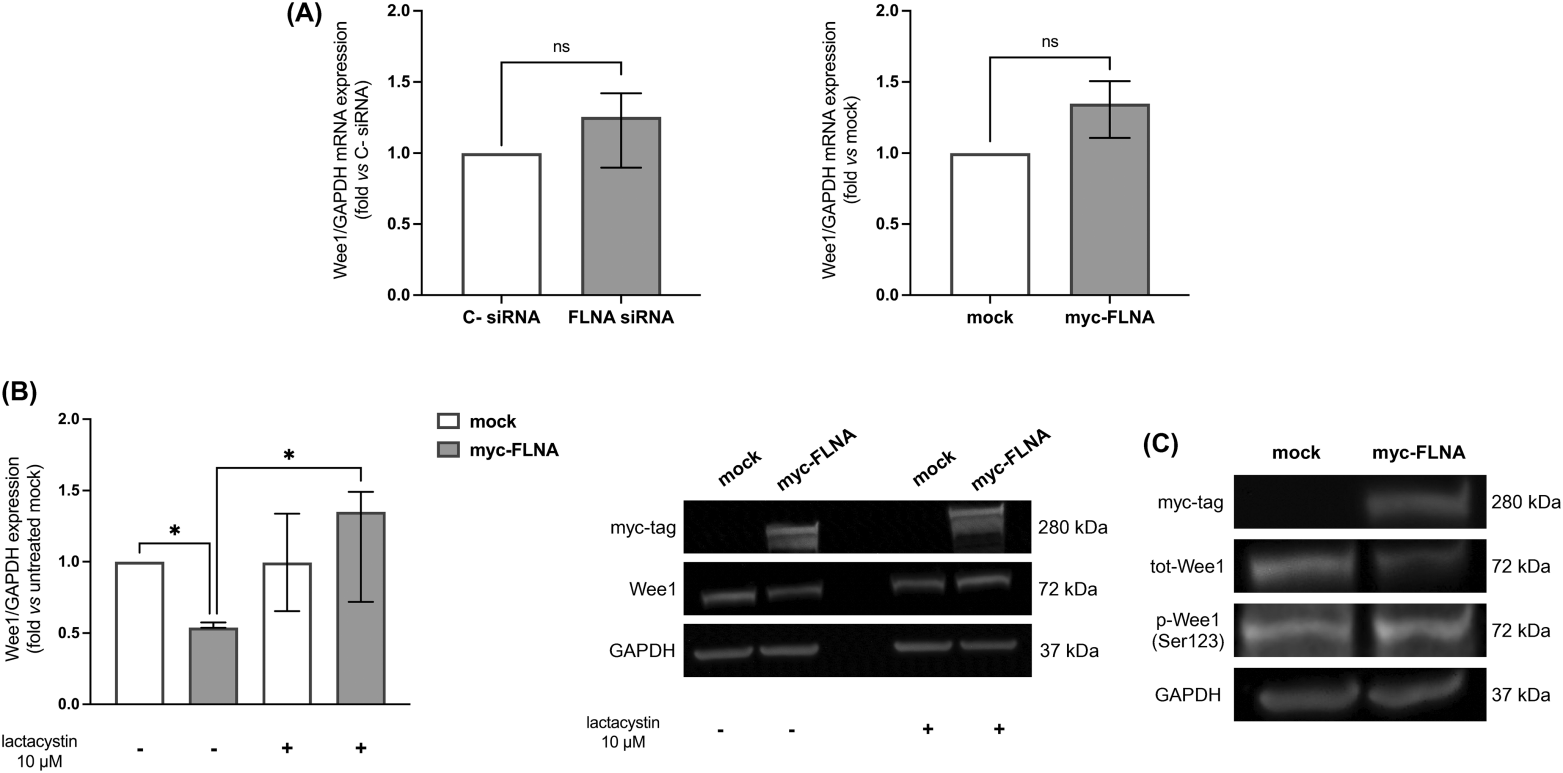
Lactacystin treatment reverted Wee1 depletion in FLNA‐transfected in MUC‐1 cell line. (A) MUC‐1 cells were transfected with FLNA siRNA or C‐ siRNA for 6 days, and with myc‐tagged FLNA plasmid for 72 h, and then RNA was extracted. The graphs show expression levels of Wee1 transcript measured by qRT‐PCR after FLNA genetic silencing and FLNA overexpression normalized to GAPDH (median and IQR of at least 3 independent experiments). Mann–Whitney test. (B) MUC‐1 cells were transiently transfected with myc‐tagged FLNA plasmid for 72 h, and then incubated with lactacystin 10 μM for 20 h. Mock‐transfected cells were used as negative controls. The graph shows densitometric analysis of Wee1 normalized to GAPDH (median and IQR of at least 3 independent experiments), expressed as fold vs mock (untreated). Representative immunoblots are shown. *p < 0.05 of untreated myc‐FLNA vs untreated mock, and of lactacystin‐treated myc‐FLNA vs untreated myc‐FLNA. Kruskal–Wallis test with Dunn's post‐hoc test. (C) MUC‐1 cells were transiently transfected with myc‐tagged FLNA plasmid for 72 h. Mock‐transfected cells were used as negative control. Representative immunoblots are shown. Due to the poor quality of p‐Wee1 (Ser123) antibody, densitometrical analysis could not be performed. Three independent experiments were carried out.

To study the mechanism underlying FLNA regulation of Wee1 expression, we tested possible alterations in Wee1 transcript levels after FLNA knockdown or overexpression. Our qRT‐PCR data showed that neither FLNA silencing nor FLNA overexpression induced a statistically significant change in Wee1 transcript levels (Figure 3A). Thus, we hypothesized that FLNA might promote Wee1 protein degradation, which occurs through the ubiquitin‐proteasome pathway. To this aim, FLNA‐transfected cells were incubated with lactacystin, a proteasome inhibitor. As expected, Wee1 expression was reduced in cells overexpressing FLNA (0.54‐fold[0.03], *p* < 0.05 vs mock‐transfected cells), while no significant changes in the expression of Wee1 were detected in FLNA‐overexpressing MUC‐1 after treatment with lactacystin (Figure 3B). As Wee1 degradation is initiated by its CDK1‐mediated phosphorylation at Ser123, we next evaluated the effect of FLNA transfection on Wee1 Ser123 phosphorylation. An increased phosphorylation of this residue was found in FLNA‐overexpressing cells compared to mock‐transfected ones (Figure 3C). Taken together, these data suggested that FLNA has a role in targeting Wee1 for proteasomal degradation.

### 3.4 Inhibition of Wee1 by AZD1775 treatment, as well as its genetic silencing, reduced cell proliferation and increased apoptosis in ACC cells

**FIGURE 4:**
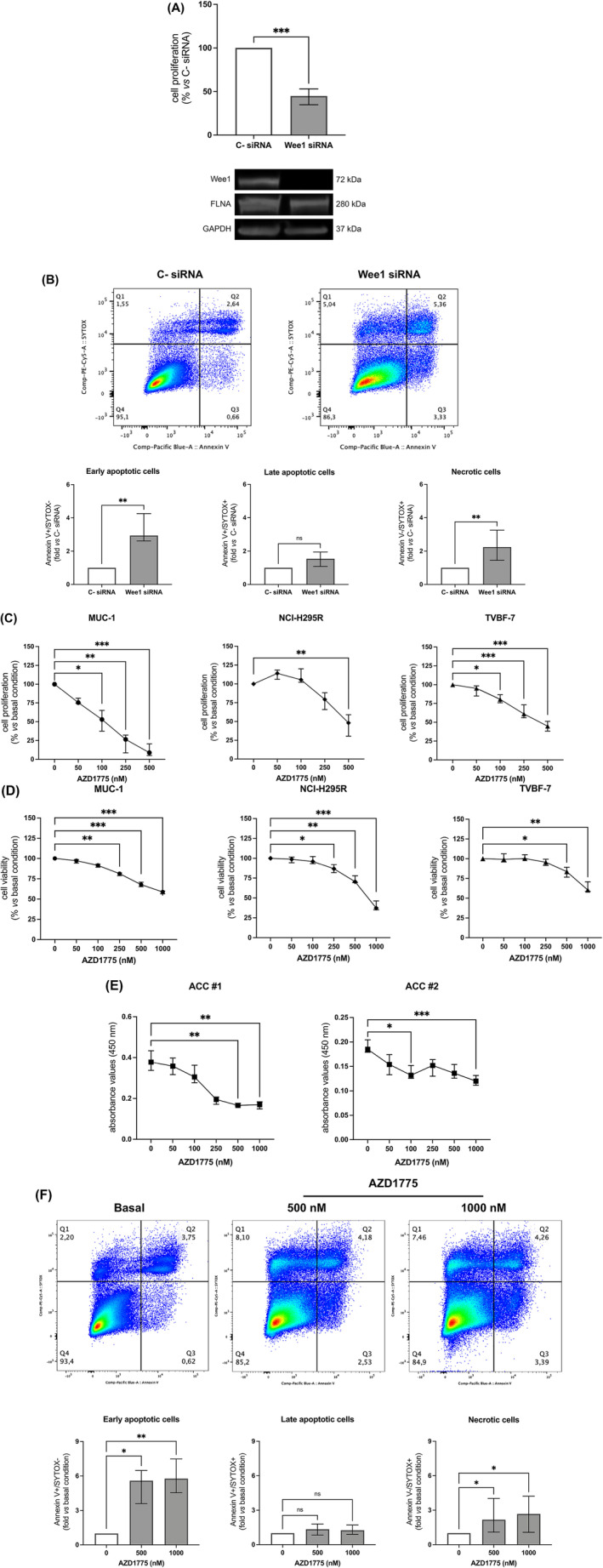
Effects of Wee1 knockdown and Wee1 kinase inhibitor AZD1775 on ACC cells. (A) Proliferation assay in Wee1‐silenced MUC‐1 cells. Cells were transfected with Wee1 siRNA or negative control (C‐) siRNA for 72 h. Cells were incubated with BrdU for 2 h, and its incorporation into newly synthetized DNA was measured (median and IQR of at least 3 independent experiments). Representative immunoblots of Wee1 silencing are shown. ***p < 0.001 vs C‐ siRNA. Mann–Whitney test. (B) Detection of MUC‐1 cell apoptosis by Pacific Blue Annexin V/SYTOX AAdvanced Apoptosis kit. Unlabelled cells were used as negative control. After 72 h of transfection, the percentage of Annexin V+ and SYTOX+ cell fractions were measured. Representative flow cytometric plots are shown. Early apoptotic, late apoptotic, and necrotic cells are plotted graphically and are expressed as fold vs C‐ siRNA (median and IQR of at least 3 independent experiments). **p < 0.01 vs C‐ siRNA. Mann–Whitney test. (C) Cell proliferation assay in MUC‐1, NCI‐H295R, and TVBF‐7 cell lines. Subconfluent cells were stimulated with increasing concentrations of AZD1775 for 72 h. After 2 h of incubation with BrdU, its incorporation into newly synthetized DNA was measured (median and IQR of at least 3 independent experiments, respectively). *p < 0.05; **p < 0.01; ***p < 0.001 vs basal condition. Friedman test with Dunn's post‐hoc test. (D) Cell viability assay in MUC‐1, NCI‐H295R, and TVBF‐7 cell lines. Subconfluent cells were stimulated with increasing concentrations of AZD1775 for 72 h. MTT assay was used to verify cell viability in response to AZD1775 treatment (median and IQR of at least 3 independent experiments, respectively). *p < 0.05; **p < 0.01; ***p < 0.001 vs basal condition. Friedman test with Dunn's post‐hoc test. (E) Cell proliferation assay in two different patient‐derived primary cultures of ACC (ACC #1 and ACC #2). Subconfluent cells were stimulated with increasing concentrations of AZD1775 for 72 h. After 24 h of incubation with BrdU, its incorporation into newly synthetized DNA was measured (median and IQR of 5 replicates for each condition). *p < 0.05; **p < 0.01; ***p < 0.001 vs basal absorbance value. Kruskal–Wallis test with Dunn's post‐hoc test. (F) Detection of MUC‐1 cell apoptosis by Pacific Blue Annexin V/SYTOX AAdvanced Apoptosis kit. Unlabelled cells were used as negative control. After 72 h of treatment with AZD1775, the percentage of Annexin V+ and SYTOX+ cell fractions were measured. Representative flow cytometric plots are shown. Early apoptotic, late apoptotic, and necrotic cells are plotted graphically and are expressed as fold vs basal condition (median and IQR of at least 3 independent experiments). *p < 0.05; **p < 0.01 vs basal condition. Friedman test with Dunn's post‐hoc test.

Next, Wee1 genetic silencing was first performed to evaluate the impact of Wee1 on ACC cell growth. Compared to cells transfected with the negative control siRNA, Wee1 knockdown significantly suppressed MUC‐1 cell proliferation (−55.1[18.2%], *p* < 0.001). Wee1 silencing did not affect the expression levels of FLNA (Figure 4A). Furthermore, an increase in early apoptotic and necrotic cell populations (2.94‐fold[1.64], and 2.24‐fold[1.80] vs C‐ siRNA, *p* < 0.01, respectively), but not of late apoptotic, was also observed in MUC‐1 cells transfected with Wee1‐specific siRNA (Figure 4B). Altogether, these data confirmed that Wee1 inhibition not only suppresses cell proliferation, but also induces cell death mechanisms, probably as a consequence of progressive DNA damage accumulation. To test the effects of pharmacological Wee1 inhibition on cell proliferation, viability and apoptosis, MUC‐1, NCI‐H295R, and TVBF‐7 cells were incubated with increasing concentrations of the specific Wee1 inhibitor AZD1775. After 72 h of treatment, a significant dose‐dependent reduction in cell proliferation was observed in all ACC cell lines (*MUC‐1*: −91.1(15.3%), *p* < 0.001; *NCI‐H295R*: −51.8(28.5%), *p* < 0.01; *TVBF‐7*: −52.7(11.3%), *p* < 0.001 vs basal at 500 nM) (Figure 4C). Moreover, we observed a reduction in cell viability (*MUC‐1*: −41.3(3.2%), *p* < 0.001; *NCI‐H295R*: −62.7(11.2%), *p* < 0.001; *TVBF‐7*: −39.6(12.1%), *p* < 0.01 vs basal at 1000 nM) (Figure 4D). A significant decrease of cell proliferation after AZD1775 incubation was also reported in primary cultured cells deriving from two different surgically removed ACC (Figure 4E). Furthermore, as observed after Wee1 silencing, treatment with AZD1775 increased both the early apoptotic (5.60‐fold[2.88], *p* < 0.05, and 5.76‐fold[2.93], *p* < 0.01 vs basal, at 500 and 1000 nM, respectively) and necrotic (2.17‐fold[2.93], *p* < 0.05, and 2.67‐fold[3.14], *p* < 0.05 vs basal, at 500 and 1000 nM, respectively) cell subpopulations (Figure 4F).

### 3.5 AZD1775 treatment altered the cell cycle pattern and induced DNA damage in MUC‐1 cells

**FIGURE 5:**
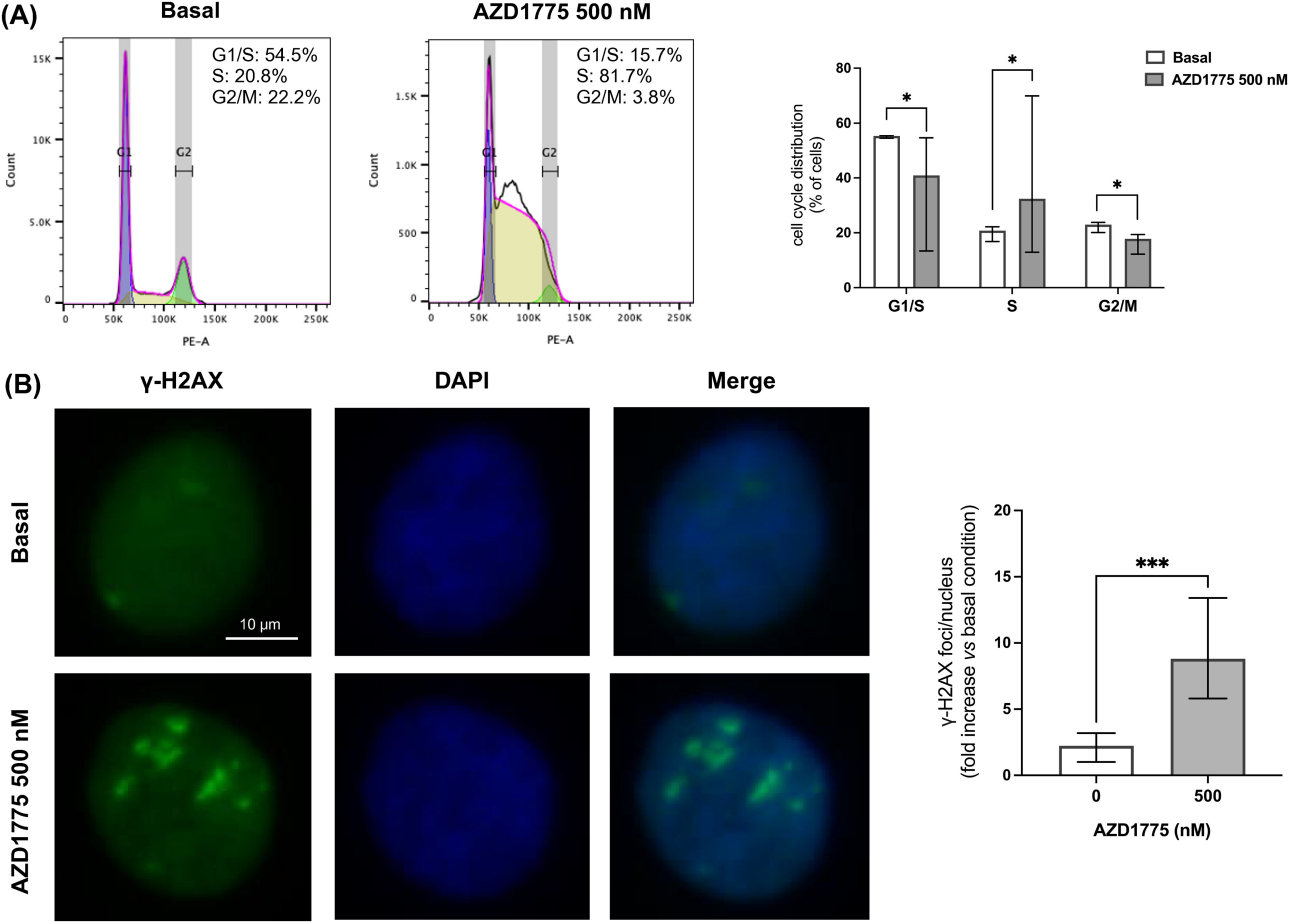
AZD1775 induced cell cycle dysregulation and DNA damage in MUC‐1 cells. (A) AZD1775 induced S phase accumulation, and G1/S and G2/M reduction in MUC‐1 cell line. Subconfluent MUC‐1 cells were treated with AZD1775 500 nM for 72 h, and DNA content was then evaluated by flow cytometry after propidium iodide (PI) staining. The graph shows the % of cells in each stage of the cell cycle (median and IQR of at least 3 independent experiments). *p < 0.05 vs basal condition. Wilcoxon test. (B) γ‐H2AX immunofluorescence staining on MUC‐1 cells untreated and treated with AZD1775 500 nM for 6 h. Nuclei were counterstained with DAPI to allow visualization. Representative images are shown. γ‐H2AX were quantified as foci/nucleus and at least 100 nuclei were scored for each experiment (median and IQR of at least 3 independent experiments). ***p < 0.001 vs basal condition. Wilcoxon test.

Given the role of Wee1 as a guardian of both S‐ and G2/M checkpoints, we then examined in MUC‐1 cells the effect of AZD1775 on cell cycle distribution. AZD1775 treatment strongly increased the amount of S phase cells (19.5%[6.0] vs 68.7%[26.2], *p* < 0.05 vs basal), and it also reduced the portion of cells stalled at G2/M (22.6[4.8] vs 6.7[8.7], *p* < 0.05 vs basal) and G1/S checkpoints (55.3[1.1] vs 17.2[15.5], *p* < 0.05 vs basal) (Figure 5A). Being the induction of DNA damage one of the major consequences of Wee1 inhibition, we also evaluated the expression of the replication stress marker γ‐H2AX. Treatment of MUC‐1 with AZD1775 markedly increased the accumulation of γ‐H2AX foci (9.7‐fold[8.1] vs 2.2‐fold[2.2], *p* < 0.001 vs basal), which are representative of double‐strand breaks (DSBs) formation. Altogether, these findings confirmed that Wee1 inhibition disrupts S‐ and G2/M checkpoints in response to replication stress, leading to increased DNA damage accumulation that finally leads to cell apoptosis (Figure 5B).

### 3.6 FLNA knockdown potentiated AZD1775 anti‐proliferative and pro‐apoptotic effects

**FIGURE 6:**
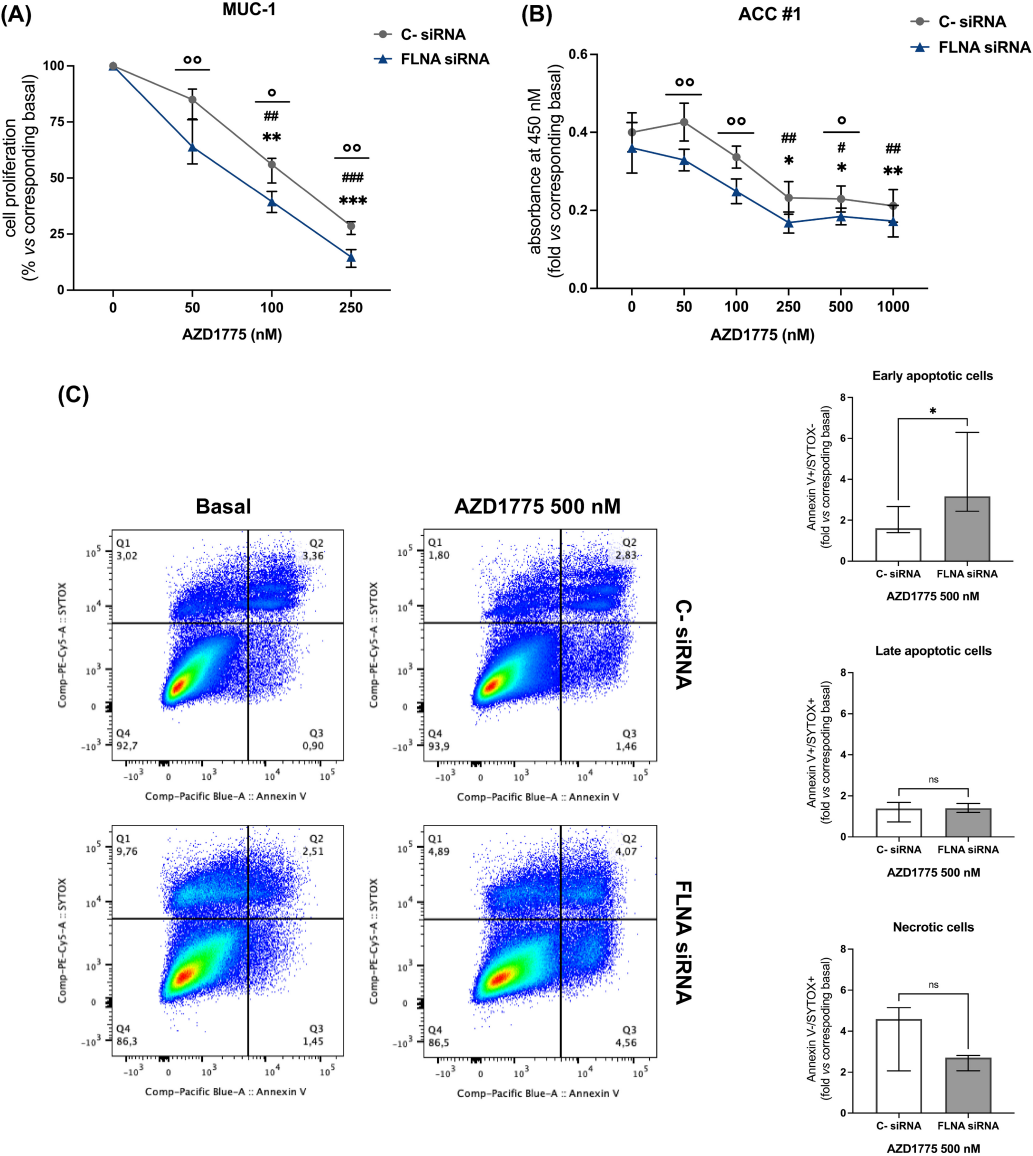
Effects of FLNA knockdown on cell proliferation of MUC‐1 and one patient‐derived primary cultured ACC cells, and on MUC‐1 apoptosis. (A) Cell proliferation assay in MUC‐1 silenced for FLNA. Subconfluent cells were silenced with FLNA siRNA for 6 days, and then stimulated with increasing concentrations of AZD1775 for 72 h. After 2 h of incubation with BrdU, its incorporation into newly synthetized DNA was measured (median and IQR of at least 3 independent experiments, respectively). **p < 0.01; ***p < 0.001 of treated C‐ siRNA vs basal C‐ siRNA. Friedman test with Dunn's post‐hoc test. ^##^ *p* < 0.01; ^###^ *p* < 0.001 of treated FLNA siRNA vs basal FLNA siRNA. Friedman test with Dunn's post‐hoc test.°*p* < 0.05;°°*p* < 0.01 of FLNA siRNA vs C‐ siRNA at the same drug concentration. Mann–Whitney test. (B) Cell proliferation assay in one patient‐derived primary culture of ACC silenced for FLNA. Subconfluent cells were silenced with FLNA siRNA for 6 days, and then stimulated with increasing concentrations of AZD1775 for 72 h. After 24 h of incubation with BrdU, its incorporation into newly synthetized DNA was measured (median and IQR of 5 replicates for each condition). *p < 0.05; **p < 0.01 of treated C‐ siRNA vs basal C‐ siRNA. Kruskal–Wallis test with Dunn's post‐hoc test. ^#^ *p* < 0.05; ^##^ *p* < 0.01 of treated FLNA siRNA vs basal FLNA siRNA. Kruskal–Wallis test with Dunn's post‐hoc test.°*p* < 0.05;°°*p* < 0.01 of FLNA siRNA vs C‐ siRNA at the same drug concentration. Mann–Whitney test. (C) MUC‐1 cell apoptosis was detected by Pacific Blue Annexin V/SYTOX AAdvanced Apoptosis Kit. Unlabelled cells were used as negative control. MUC‐1 were transfected with the FLNA‐specific siRNA for 6 days. After treatment with AZD1775, the percentage of Annexin V+ and SYTOX+ cell fractions were measured. Representative flow cytometric plots are shown. Early apoptotic, late apoptotic, and necrotic cells are plotted graphically and are expressed as fold vs C‐ siRNA (median and IQR of at least 3 independent experiments). *p < 0.05 of C‐ siRNA vs FLNA siRNA at the same drug concentration. Mann–Whitney test.

Since the loss of FLNA expression found in ACC is associated with an increase in Wee1 expression and activity, we then examined whether the efficacy of AZD1775 in decreasing cell proliferation was affected by FLNA expression levels. We found an increased efficacy of AZD1775 in reducing cell proliferation in MUC‐1 silenced for FLNA (−84.8(8.0%) for FLNA siRNA vs −71.9(6.4%) for C‐ siRNA, *p* < 0.01 at 250 nM) (Figure 6A), and similar results were obtained in one patient‐derived primary cultured ACC cells (Figure 6B). Analogously, FLNA silencing potentiated the pro‐apoptotic effect of AZD1775 in MUC‐1 by increasing the early apoptotic cell subpopulation (3.15‐fold[2.6] for FLNA siRNA vs 1.78‐fold[1.08] for C‐ siRNA, *p* < 0.05 at 500 nM; Figure 6C).

## 4 DISCUSSION

Integrated pan‐genomic molecular profiling studies have provided great insight into the pathogenesis of ACC by revealing recurrent alterations in genes mainly involved in the Wnt/β‐catenin pathway, cell cycle regulation, and chromatin remodelling.^33^, ^34^ However, dissecting the complex molecular mechanisms involved in ACC pathogenesis and progression still remains a challenge. Since alterations in cell cycle‐related genes have been reported as highly important ACC drivers, expression patterns of G2/M phase regulators as CDK1, cyclin B1, polo‐like kinase 1 (PLK1), Cdc25C, and DNA Topoisomerase 2 Alpha (TOP2A), have been extensively studied and found upregulated in ACC compared to ACA and NAG.^6^, ^35^, ^36^, ^37^, ^38^, ^39^ In this work, we found an overexpression in ACC of Wee1 nuclear kinase, another leading regulator of G2/M checkpoint transition, suggesting that Wee1 may represent a new promising druggable target. Moreover, we showed the anti‐tumour effects of the specific Wee1 inhibitor AZD1775 as a single agent in ACC cells. The absence of the cytoskeletal protein filamin A (FLNA), found in the majority of ACC, is associated to a more aggressive tumour behaviour.^7^ Since FLNA knockdown has been demonstrated to induce an increase in Wee1,^11^ we investigated the role played by FLNA in regulating Wee1 expression and the response to AZD1775.

Three human ACC cell lines, either originating from a primary tumour (NCI‐H295R) or an ACC metastasis (MUC‐1 and TVBF‐7) were used. Specifically, MUC‐1 were obtained from a distant ACC neck metastasis, while TVBF‐7 were established from a perirenal lymph‐node metastasis. All of them harbours cancer‐associated mutations^40^ but, contrarily to TVBF‐7, NCI‐H295R and MUC‐1 cells carry mutations in *TP53* locus, which has been recognized as one of the key ACC driver genes. Therefore, having a different genetic background, they reflect the heterogeneity typical of ACC, but they also allow to predict drug response according to specific patient sub‐type characteristics.^40^ Both ACC cell lines of metastatic origin reflect the clinically low response to EDP‐M scheme.^31^, ^32^ Particularly, MUC‐1 cells were reported to be highly resistant to a wide range of therapies,^29^, ^41^, ^42^, ^43^, ^44^ thus they represent an extremely useful model to look for novel therapies for advanced ACC. In addition to cell lines, patient‐derived primary ACC cell cultures were also used in this study.

Protein expression analysis in 6 patients‐derived ACC, 8 ACA, and 8 NAG tissue samples revealed a Wee1 upregulation in ACC compared to NAG. Whereas a reduced expression of FLNA protein was found in ACC compared to NAG. Upregulation of Wee1 has been found in several tumours, and it is frequently associated to a poor prognosis.^16^, ^17^, ^18^, ^19^ On the contrary, a reduced expression of FLNA protein was found in ACC compared to NAG, as we have previously reported.^7^

The subsequent investigation of FLNA and Wee1 protein levels in ACC cell lines revealed that all of them express both proteins at different levels. Even in this case, the expression of FLNA and Wee1 expression seemed to be regulated by an inversely proportional relationship, with MUC‐1 showing the highest FLNA and the lowest Wee1 expression, while NCI‐H295R and TVBF‐7 expressing very low FLNA and high Wee1 protein levels. Due to the absence of a FLNA‐lacking ACC cellular model, genetic FLNA silencing was initially carried out in all cell lines to investigate a possible causal association between low FLNA and high Wee1 expression detected in ACC. An efficient knockdown was just shown in MUC‐1 cell line, which was then selected to study the functional impact of FLNA silencing on Wee1 protein and its main downstream targets expression. Transfection of FLNA siRNA increased Wee1 protein levels in both MUC‐1 and one patient‐derived ACC primary cultured cells. Moreover, a boosted Wee1 activity, as suggested by higher levels of phosphorylated CDK1 and cyclin B1, was reported in MUC‐1 cells after FLNA silencing. Our results were broadly in line with that of *Lian* et al., who reported that FLNA regulated Wee1 expression and stability, as well as cyclin B1 degradation by affecting the status of CDK1 Tyr15 phosphorylation in neural progenitors.^11^ This mechanism was further clarified by transfection of myc‐tagged FLNA plasmid in MUC‐1. In this case, there was a significant lowering of Wee1, phosphorylated CDK1 and cyclin B1 expression associated with FLNA overexpression, confirming that, in this specific cell line, the expression of Wee1 and that of the proteins involved in its specific pathway strictly depends on FLNA levels.

We then speculated that FLNA might indirectly promote Wee1 proteasomal degradation. This hypothesis was supported by the observation that treatment with the proteasomal inhibitor lactacystin reverted Wee1 reduction in MUC‐1 overexpressing FLNA. Moreover, it is notable that FLNA overexpression increased levels of phosphorylated Wee1 at Ser123, a signal that directs Wee1 to proteasomal degradation.^13^ Since it has been demonstrated that cyclin B1/CDK1 binds to FLNA, we can speculate that FLNA acts as a scaffold allowing CDK1 to phosphorylate Wee1 at Ser123, thus promoting its proteasomal degradation.^45^

Transfection of MUC‐1 with siRNA targeting Wee1 resulted in a strong reduction in cell proliferation and an increase in apoptosis, consistent with the finding that dysregulated Wee1 function has a role in tumour progression.^18^, ^19^, ^20^, ^21^ Of note, siRNA‐mediated knockdown of Wee1 did not impact FLNA protein levels, suggesting that these two proteins are not mutually regulated.

We next assessed cell proliferation and viability in all ACC cell lines following AZD1775 treatment, and we showed a significant dose‐dependent reduction in both cell proliferation and viability in all of them. It is worth saying that we found no relation between cells carrying a *TP53* mutation and sensitivity to AZD1775 treatment. Indeed, MUC‐1 (*TP53* ^mut^) showed a very high responsiveness to Wee1 inhibition on cell proliferation even at very low drug dosages, while no considerable differences were detected between NCI‐H295R (*TP53* ^mut^) and TVBF‐7 (*TP53* ^wt^) responses to AZD1775. Inhibition of cell viability by AZD1775 was stronger in NCI‐H295R, while a similar response to Wee1 inhibition was reported in MUC‐1 and TVBF‐7 cells viability. These results are in line with previous studies that demonstrated that *TP53* mutational status is not predictive of AZD1775 response.^23^, ^24^, ^25^ A significant dose‐dependent reduction in cell proliferation was also shown in primary cells deriving from two surgically removed ACC.

Wee1 inhibition by AZD1775 has been shown to cause increased DNA damage and induction of apoptosis in a variety of cancers.^23^, ^24^, ^25^ Consistent with these findings, we reported that MUC‐1 exposure to AZD1775 induced a dose‐dependent increase in early apoptotic and necrotic cell subpopulations. As expected, a highly altered cell cycle distribution pattern was found after AZD1775 treatment. Particularly, we reported a strong increase in cells at S phase and a reduction in cells halted at G1/S and G2/M checkpoints. Moreover, following AZD1775 exposure, we showed elevated levels of the DNA damage marker γ‐H2AX caused by replication stress. Altogether, these results tie well with previous studies wherein inhibition of Wee1 was shown to abrogate G1/S checkpoint and to induce DNA damage in the S phase of the cell cycle.^46^, ^47^ Furthermore, reduction of cells in G2/M supports the mechanism of G2/M checkpoint override, resulting in premature mitosis entrance.^21^, ^48^, ^49^ Therefore, consistent to what reported by *AArts* et al.,^50^ we might speculate that Wee1 inhibition by AZD1775 forces DNA‐damaged cells arrested at S‐phase into a premature mitosis, finally resulting into cell death.

Having reported that FLNA knockdown led to high levels of Wee1 expression and activity, we moved to examine whether increased levels of its target might affect cells response to AZD1775. Our data demonstrated that high Wee1 expression levels sensitized MUC‐1 to AZD1775 anti‐tumour effects, causing a further reduction in cell proliferation and increase in apoptotic cells.

In conclusion, the data presented here together propose Wee1 inhibition as a new potential therapeutic approach for ACC, particularly for those lacking FLNA.

## AUTHOR CONTRIBUTIONS

Emanuela Esposito: Conceptualization, Data curation, Formal analysis, Investigation, Methodology, Software, Visualization, Writing‐original draft. Giusy Marra: Data curation, Investigation, Visualization. Rosa Catalano: Data curation, Formal analysis, Investigation. Sara Maioli: Formal analysis, Investigation, Software. Emma Nozza: Formal analysis, Investigation, Methodology. Anna Maria Barbieri: Investigation, Methodology, Software. Constanze Hantel: Resources, Validation, Writing—review & editing. Guido Di Dalmazi: Resources, Validation, Writing—review & editing. Sandra Sigala: Resources, Validation, Writing—review & editing. Jens Geginat: Resources, Validation, Writing—review & editing. Elisa Cassinotti: Resources, Validation, Writing—review & editing. Ludovica Baldari: Resources, Validation, Writing—review & editing. Serena Palmieri: Resources, Validation, Writing—review & editing. Alessandra Mangone: Resources, Validation, Writing—review & editing. Alfredo Berruti: Resources, Validation, Writing—review & editing. Emanuele Ferrante: Resources, Validation, Writing—review & editing. Giovanna Mantovani: Funding acquisition, Project administration, Supervision, Validation, Writing—review & editing. Erika Peverelli: Conceptualization, Funding acquisition, Methodology, Project administration, Supervision, Validation, Writing—review & editing. The study reported in the article has been performed by the authors, unless clearly specified in the text.

## FUNDING INFORMATION

This work was supported by an AIRC (Associazione Italiana Ricerca Cancro) grant to Erika Peverelli (IG 2021‐25920), by Progetti di Ricerca di Interesse Nazionale (PRIN) grants to Erika Peverelli (2022CZR88M and P20227KXJK), and by Ricerca Corrente Funds from the Italian Ministry of Health to Fondazione IRCCS Ca' Granda Ospedale Maggiore Policlinico of Milan.

## CONFLICT OF INTEREST STATEMENT

All authors have declared no potential conflicts of interest.

## ETHICS STATEMENT

This study was approved by the Ethical Committee of CE‐AVEC (105/2017/U/Tess) and by Milano Area 2 Ethical Committee (554_2022bis), and patients who underwent adrenalectomy gave informed consent to the use of their tumour samples and clinical information.

## Data Availability

The data that support the finding of this study are available from the corresponding authors (E. P., G. M.), upon reasonable request.
